# From ELISA to Immunosorbent Tandem Mass Spectrometry Proteoform Analysis: The Example of CXCL8/Interleukin-8

**DOI:** 10.3389/fimmu.2021.644725

**Published:** 2021-03-11

**Authors:** Mieke Metzemaekers, Sara Abouelasrar Salama, Jennifer Vandooren, Anneleen Mortier, Rik Janssens, Sofie Vandendriessche, Eva Ganseman, Erik Martens, Mieke Gouwy, Barbara Neerinckx, Patrick Verschueren, Lien De Somer, Carine Wouters, Sofie Struyf, Ghislain Opdenakker, Jo Van Damme, Paul Proost

**Affiliations:** ^1^Laboratory of Molecular Immunology, Department of Microbiology, Immunology and Transplantation, Rega Institute, Katholieke Universiteit Leuven, Leuven, Belgium; ^2^Laboratory of Immunobiology, Department of Microbiology, Immunology and Transplantation, Rega Institute, Katholieke Universiteit Leuven, Leuven, Belgium; ^3^Department of Development and Regeneration, Skeletal Biology and Engineering Research Center, Katholieke Universiteit Leuven, Leuven, Belgium

**Keywords:** chemokine, CXCL8, neutrophil, ELISA, proteolysis, posttranslational modification, arthritis, top-down mass spectrometry

## Abstract

With ELISAs one detects the ensemble of immunoreactive molecules in biological samples. For biomolecules undergoing proteolysis for activation, potentiation or inhibition, other techniques are necessary to study biology. Here we develop methodology that combines immunosorbent sample preparation and nano-scale liquid chromatography—tandem mass spectrometry (nano-LC-MS/MS) for proteoform analysis (ISTAMPA) and apply this to the aglycosyl chemokine CXCL8. CXCL8, the most powerful human chemokine with neutrophil chemotactic and –activating properties, occurs in different NH_2_-terminal proteoforms due to its susceptibility to site-specific proteolytic modification. Specific proteoforms display up to 30-fold enhanced activity. The immunosorbent ion trap top-down mass spectrometry-based approach for proteoform analysis allows for simultaneous detection and quantification of full-length CXCL8(1-77), elongated CXCL8(-2-77) and all naturally occurring truncated CXCL8 forms in biological samples. For the first time we demonstrate site-specific proteolytic activation of CXCL8 in synovial fluids from patients with chronic joint inflammation and address the importance of sample collection and processing.

## Introduction

Neutrophils are the most abundant leukocyte type in human blood (1). These innate immune cells are endowed with a comprehensive anti-microbial machinery and are usually the first responders to infection or tissue injury (2). Adequate spatiotemporal trafficking and activation of neutrophils are essential to guarantee immune surveillance and to prevent inappropriate immune activation leading to tissue damage. Chemokines play a central role in this process (3). These low molecular mass proteins function primarily by activation of dedicated heptahelical G protein-coupled receptors (GPCRs) (4, 5). However, the precise chemokine activity and availability *in vivo* are the labyrinthine outcome of multiple regulatory mechanisms with a significant role for posttranslational modifications (6–9). Mature secreted chemokines are susceptible to site-specific proteolysis, citrullination, nitration and glycosylation, with modification- and ligand-dependent consequences for their biological functions [reviewed in (6, 9–11)].

CXCL8 or interleukin (IL-) 8 is the most powerful neutrophil-attracting chemokine in humans. CXCL8 is a small protein without glycosylation (aglycosyl) and is produced by virtually any cell type upon appropriate stimulation. It has been widely implicated in diseases including autoimmune disorders and cancer (12, 13). CXCL8 was described for the first time in the late 80s by four independent research groups (14–18). Upon its discovery, it was clear that CXCL8 displays a remarkable degree of natural NH_2_-terminal sequence heterogeneity, due to its sensitivity to proteolytic modification in particular (15, 19). Follow-up research revealed that, in general, NH_2_-terminally shortened CXCL8 proteoforms exhibit a superior capacity to chemo-attract and activate neutrophils (up to 30 times more potent) as compared to full-length CXCL8(1-77) on condition that the conserved ELR motif remains intact (20–26). In addition, elongated CXCL8(-2-77)—that presumably originates from alternative splicing of the signal peptide—has a moderately increased biological activity as compared to CXCL8(1-77), but is less efficiently processed to the more potent form CXCL8(6-77) (27). Enzymes responsible for CXCL8 cleavage include plasmin, thrombin, CD13, matrix metalloproteinase (MMP)-1, MMP-8, MMP-9, MMP-13, MMP-14 and cathepsins B, G, K, L, and S. They are usually upregulated in pathological conditions, supporting the idea that CXCL8 processing becomes predominantly relevant during inflammation. This further sparked our interest to develop methods sensitive enough to quantify CXCL8 proteoforms in small-sized human samples.

The currently available standard immunoassays do not discriminate between authentic and processed chemokines with divergent biological activities. Indeed, most commercially available antibodies recognize the different forms of a specific chemokine with similar efficiency. Western blot approaches may point toward modifications with major effects on the molecular mass of the protein involved, but cannot detect subtle proteolysis nor reveal the exact identity and activation state of the processed molecule. Moreover, physical separation of chemokine forms is challenging since proteins with minimal structural differences co-elute from conventional columns used in ion exchange and reversed phase (RP) chromatography (20).

**Figure 1:**
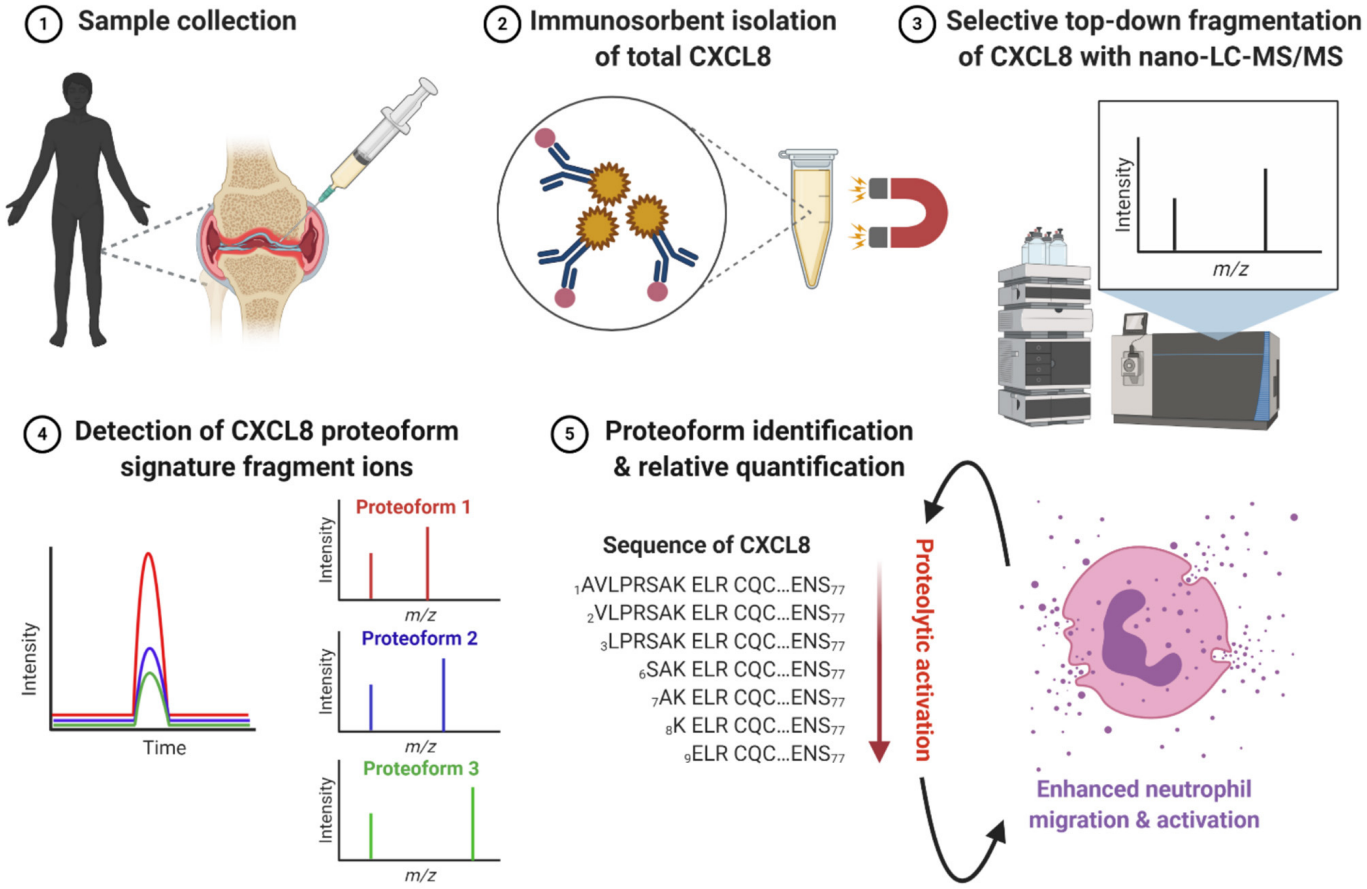
Workflow for immunosorbent tandem mass spectrometry proteoform analysis (ISTAMPA). (1) Synovial fluids are collected from the inflamed joints of patients with arthritis. (2) Total CXCL8 is extracted from patient samples by immunosorbent purification using antibody-coupled magnetic beads. (3) Isolated CXCL8 proteoforms are subjected to analysis by nano-UPLC-MS/MS. (4, 5) The detection and quantification of signature fragment ions, generated by top-down collision-induced dissociation (CID) of a pre-selected precursor ion, at the expected elution time point, strongly indicates the presence of a specific CXCL8 proteoform. In general, truncated CXCL8 proteoforms display an enhanced capacity to induce neutrophil migration and activation. This figure is created with BioRender software.

Here, we introduce a straightforward *i*mmuno*s*orbent nano-scale liquid chromatography – *ta*ndem *m*ass spectrometry (nano-LC-MS/MS)-based methodology for *p*roteoform *a*nalysis called ISTAMPA and apply this to CXCL8 (Figure 1). This top-down approach allows for detection and quantification of authentic CXCL8(1-77), elongated CXCL8(-2-77) and all naturally occurring truncated and activated CXCL8 proteoforms i.e., CXCL8(2-77), CXCL8(3-77), CXCL8(6-77), CXCL8(7-77), CXCL8(8-77), and CXCL8(9-77) in biological samples. Moreover, this study is the first to report site-specific cleavage of CXCL8 in synovial fluids from patients with rheumatoid arthritis (RA) and juvenile idiopathic arthritis (JIA), thereby opening a window of oppurtunitiy for identification of disease biomarkers and potential therapeutic targets.

## Methods and Materials

### Chemical Synthesis of CXCL8 Proteoforms

CXCL8(-2-77), CXCL8(1-77), CXCL8(2-77), CXCL8(3-77), CXCL8(6-77), CXCL8(7-77), CXCL8(8-77), and CXCL8(9-77) were chemically synthesized on an Activotec P11 automated peptide synthesizer (Activotec, Cambridge, U.K.) based on N-(9-fluorenyl) methoxycarbonyl (Fmoc) chemistry as described (28). RP – high performance liquid chromatography (HPLC) was used to purify synthesized proteins to homogeneity (Proto 300 C4 column; 150 ×4.6 mm, Higgins Analytical Inc., Mountain View, CA). Elution was performed with an acetonitrile gradient in 0.1% (*v/v*) trifluoroacetic acid (TFA), with two percent of the effluent being used for analysis with online electrospray ionization – ion trap mass spectrometry (AmaZon SL mass spectrometer; Bruker Daltonics, Bremen, Germany). Homogeneous CXCL8 proteins were folded into their correct configurations as reported previously (28). The purity and concentration of synthesized and correctly folded CXCL8 forms was confirmed by ion trap mass spectrometry and specific sandwich ELISAs, respectively.

### Patients

Patients were recruited at the University Hospital Leuven after informed consent according to the ethical guidelines of the Declaration of Helsinki. Parents or legal guardians signed the informed consent on behalf of children. Synovial fluid was collected only if joint aspiration was required for treatment. Synovial fluids were collected in BD vacutainer tubes containing ethylenediaminetetraacetic acid (EDTA) (BD Biosciences, East Rutherford, NJ). The Ethics Committee of the University Hospital Leuven approved experiments involving human samples (S59874 and ML1814).

### Antibody Binding Assay

CXCL8 forms were immobilized on 96-well plates through overnight incubation at 4°C. Three washing steps with [PBS + 0.05% (*v/v*) Tween-20] were performed and plates were blocked with 0.1% (*w/v*) casein during 1 h at 37°C. After three washing steps, captured CXCL8 forms were detected using biotinylated polyclonal rabbit anti-human CXCL8 (500-P28BT; PeproTech, Rocky Hill, NJ), raised against recombinant human CXCL8(6-77), combined with peroxidase-conjugated streptavidine (R&D Systems, Minneapolis, MN). Finally, detection was obtained with a peroxidase substrate solution composed of 0.1 M citrate (pH 4.9) and 0.004% (*v/v*) H_2_O_2_, and containing 0.42 mM 3,3′,5,5′-tetramethylbenzidine (TMB; Sigma Aldrich, Saint Louis, MO). Conversion of TMB was quantified via optical density (OD) measurements at 450 nm.

### Surface Plasmon Resonance

Polyclonal rabbit anti-human CXCL8 (60 μg/ml; *vide supra*) was immobilized on a carboxyl sensor (Nicoya, Kitchener, ON, Canada) using an OpenSPR benchtop surface plasmon resonance (SPR) instrument (Nicoya). The sensor was blocked with 0.1 M ethanolamine (pH 8.5). Serial dilutions of CXCL8(1-77) and CXCL9(9-77) were loaded in [10 mM HEPES + 500 mM NaCl + 0.5% (*w/v*) bovine serum albumine + 0.25% (*v/v*) Tween-20] (pH 7.4) at a flow rate of 20 μL/min. Regeneration was performed with 20 mM Gly (pH 2.6). Results were analyzed with TraceDrawer software (TraceDrawer, Uppsala, Sweden).

### Immunosorbent Isolation of CXCL8 From Synovial Fluids

For each sample, 5 μg biotinylated polyclonal rabbit anti-human CXCL8 (*vide supra*) was coupled to 25 μl streptavidin-coated magnetic particles (Dynabeads™ M-280 Streptavidin, Thermo Scientific, Waltham, MA) during 30 min at room temperature (RT) under continuous rotation. Antibody-labeled beads were washed four times with 0.5 ml PBS using a DynaMag 2 magnet (Thermo-Scientific) and incubated with 0.5 ml synovial fluid or with 0.5 ml human plasma enriched with CXCL8(1-77), CXCL8(6-77) and CXCL8(9-77) (5 ng of each form). After 30 min of incubation at room temperature under rotation, beads were washed four times with 0.5 ml PBS and CXCL8 proteoforms were eluted with 0.1 M glycine pH 2.8 (elution volume of 20 μl). Samples were directly loaded in a cooled autosampler (5°C) and analyzed by nano-LC-MS/MS. To investigate the kinetics of CXCL8 processing in the presence of synovial fluids, exogenous CXCL8(1-77) (500 ng) was subjected to human synovial fluid (20 μL) for a period of 0, 3, 6, 12 or 20 h and the same procedure was followed.

### Production of Natural CXCL8 by Osteosarcoma Cells (MG-63)

The osteosarcoma cell line MG-63 was grown in Eagle's minimum essential medium and stimulated with the synthetic double stranded RNA poly rI:rC (50 μg/ml; P-L Biochemicals, Milwaukee, WI) to produce CXCL8 as described (29). To isolate CXCL8 variants, MG-63 cell culture supernatant was concentrated and partially purified using controlled pore glass (CPG) and heparin affinity chromatography (GE Healthcare; Chicago, IL). Protein elution was achieved using a NaCl gradient of 0.05–2.0 M in 50 mM Tris-HCl (pH 7.4). Heparin-Sepharose fractions containing CXCL8 immunoreactivity, demonstrated by a specific CXCL8 ELISA developed in our laboratory (20), were further purified on a C8 Aquapore RP-300 column (220 ×2.1 mm, PerkinElmer Life Sciences; Waltham, MA) by RP-HPLC (Waters 600 HPLC System: controller and solvent delivery system; Milford, MS). CXCL8 elution was achieved through an acetonitrile gradient (0–80%) in 0.1% (*v/v*) TFA/ultrapure water (pH 2.0). UV absorbance was measured at 214 nm reflecting protein concentrations. CXCL8 proteoform identification was performed by Edman degradation on the purified fractions using a PPSQ-51A protein sequencer (Shimadzu, Kioto, Japan) and via nano-LC-MS/MS.

### Nano-LC-MS/MS

Synthesized CXCL8 proteoforms (used as reference molecules for optimization of nano-LC-MS/MS parameters) or partially purified cell culture supernatants from MG-63 osteosarcoma cells were diluted in 0.1% (*v/v*) TFA prior to analysis by nano-LC-MS/MS. Elution fractions from immunomagnetic isolation of CXCL8 proteoforms from synovial fluids or plasma were analyzed as such. Samples (5 μL) were injected on an UltiMate 3000 nano-scale RP-UPLC (Thermo Scientific) equipped with an autosampler. Physical separation of proteins was realized using a PepMap 300 C4 pre-column (5 ×0.3 mm; Thermo Scientific) combined with a Proto 300 C4 column (50 ×0.15 mm; Higgins Analytical Inc.). Samples were loaded on the pre-column in 4% (*v/v*) acetonitrile in 0.1% (*v/v*) TFA and elution was performed with an acetonitrile gradient in 0.1% (*v/v*) formic acid (flow rate of 0.5 μL/min). The eluate was directly injected into an Amazon Speed ETD mass spectrometer (Bruker Daltonics) provided with captive spray ionization – ion trap technology. Collision-induced dissociation (CID) was exploited for low-energy fragmentation (MS/MS fragmentation amplitude of 1.0) of preselected precursor ions with specific m/z values ± 2 in multiple reaction monitoring (MRM) mode. Hystar 3.2 and Trap Control 8.0 software (Bruker Daltonics) was used for data collection. Results were analyzed with Compass Data Analysis 5.0 software (Bruker Daltonics).

## Results

### Development of a Nano-LC-MS/MS-Based Tool for Identification of CXCL8 Proteoforms

**Figure 2:**
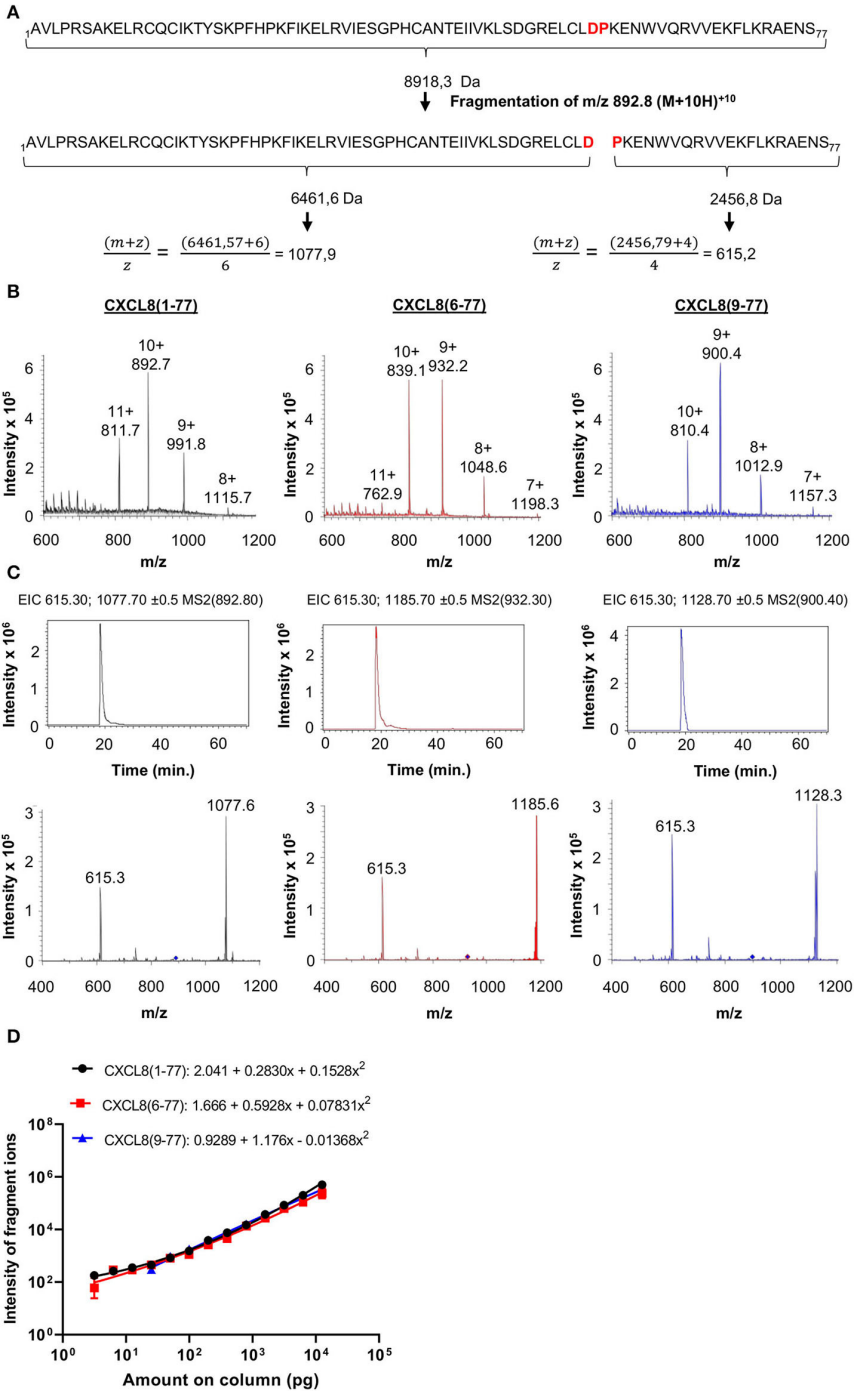
Detection of CXCL8 proteoforms by top-down tandem mass spectrometry. **(A)** Parameters are optimized to ensure that only the acid-labile Asp-Pro (DP) bond breaks during low-energy fragmentation and only two fragment ions are generated. **(B)** Mass spectra (single MS) showing the intensity of multiple charged ions of CXCL8(1-77), CXCL8(6-77) and CXCL8(9-77) as a function of their m/z values. Ions with m/z values of 892.8 [for CXCL8(1-77)], 932.3 [for CXCL8(6-77)] or 900.4 [for CXCL8(9-77)] were isolated and fragmented with low energy CID. The resulting fragmentation spectra are shown in the lower panels in **(C)**. Diamonds indicate the m/z value of the precursor ions selected for fragmentation. Extracted ion chromatograms (EIC) (top panels) show the detection of m/z values (±0.5) of the two signature fragment ions during protein elution. Relative quantification of CXCL8 forms is performed based on the intensity of the two major fragment ions in the EIC. **(D)** Dose-response curves for the simultaneous quantification of three CXCL8 forms in MRM mode (amounts ranging from 3.1 pg to 12.5 ng). Regression analysis was performed to fit curves to data. Results are represented as mean ± SEM (*n* ≥ 4).

Tandem mass spectrometry (MS/MS) has become the standard technique for protein identification in complex biological samples. Given its precision, accuracy and selectivity, we selected nano-LC-MS/MS as the method of choice for development of a novel tool that allows for identification and quantification of CXCL8 proteoforms in complex small-sized samples without the use of proteases (e.g., trypsin) during sample preparation. Authentic CXCL8(1-77), elongated CXCL8(-2-77) and all six naturally occurring truncated CXCL8 proteoforms i.e., CXCL8(2-77), CXCL8(3-77), CXCL8(6-77), CXCL8(7-77), CXCL8(8-77), and CXCL8(9-77) were chemically synthesized and used as reference molecules (protein sequences of CXCL8 forms are provided in Supplementary Figure 1). For optimization of nano-LC-MS/MS parameters, we took advantage of the fact that CXCL8 proteins contain one Asp-Pro (-D-P-) bond, i.e., the peptide bond with the highest sensitivity to acidic hydrolysis (Figure 2A; Supplementary Figure 1). To guarantee maximal sensitivity of the method, MS/MS parameters were optimized to ensure that only the D-P bond breaks during top-down CID (Figure 2A). This results in the generation of two signature fragment ions and, importantly, a minor loss in intensity. The rationale for this approach, as exemplified for full-length CXCL8(1-77), is shown in Figure 2A.

### Quantification of CXCL8 Proteoforms by Nano-LC-MS/MS

For each CXCL8 form, a precursor ion with specific mass-to-charge (m/z) value most suitable for fragmentation (usually the precursor ion with the highest intensity) was selected by single mass spectrometry. Mass- and fragmentation spectra of three major CXCL8 forms, i.e., full-length CXCL8(1-77) and NH_2_-terminally truncated forms CXCL8(6-77) and CXCL8(9-77), are shown in Figures 2B,C. An overview of the selected precursor ions for CXCL8(-2-77), CXCL8(1-77), CXCL8(2-77), CXCL8(3-77), CXCL8(6-77), CXCL8(7-77), CXCL8(8-77), and CXCL8(9-77), the resulting fragment ions and corresponding amino acids in the sequence of CXCL8 is provided in Table 1. The different CXCL8 proteoforms share a common, COOH-terminal fragment ion (m/z value 615.3 with 4 charges)—that can be used as an internal control—and have specific NH_2_-terminal fragment ions. The combination of detection of signature fragment ions, generated by fragmentation of a pre-selected precursor ion, at the expected elution time point, strongly indicates the presence of a specific CXCL8 proteoform. To explore whether nano-LC-MS/MS can be used for quantification of CXCL8 proteoforms, mixtures were prepared containing equal amounts of CXCL8(1-77), CXCL8(6-77), and CXCL8(9-77). We found that CXCL8 proteins can be quantified by determining the intensity of their fragment ions in the extracted ion chromatogram (EIC), that shows the detection of signature fragment ions generated by fragmentation of a specific precursor ion during protein elution. Dose-response curves demonstrating the simultaneous quantification of 3.1 pg−12.5 ng CXCL8(1-77), CXCL8(6-77), and CXCL8(9-77) in MRM mode are shown in Figure 2D. Up to five CXCL8 proteoforms can be measured simultaneously in MRM mode without loss of sensitivity. Analysis of a mixture containing CXCL8(1-77) and CXCL8(6-77) (ratio 1:4) confirmed succesful quantification if samples contain unequal amounts of CXCL8 proteoforms (Supplementary Figure 2). Finally, the dynamic range of detection was at least four orders of magnitude (Figure 2D).

**Table 1:** Site-specific fragmentation of CXCL8 proteoform ions by top-down ion trap tandem mass spectrometry at limited fragmentation energy.

**CXCL8 form**	**Selected precursor ion for fragmentation [m/z value (z)]**	**Signature fragment ions [m/z value (z)]**	**Corresponding amino acids in the sequence of CXCL8**
CXCL8(-2-77)	911.5 [10+]	615.3 [4+] 1105.8 [6+]	58-77 −2-57
CXCL8(1-77)	892.8 [10+]	615.3 [4+] 1077.7 [6+]	58-77 1-57
CXCL8(2-77)	885.7 [10+]	615.3 [4+] 1065.9 [6+]	58-77 2-57
CXCL8(3-77)	875.8 [10+]	615.3 [4+] 1049.3 [6+]	58-77 3-57
CXCL8(6-77)	932.3 [9+]	615.3 [4+] 1185.7 [5+]	58-77 6-57
CXCL8(7-77)	922.6 [9+]	615.3 [4+] 1168.3 [5+]	58-77 7-57
CXCL8(8-77)	914.6 [9+]	615.3 [4+] 1154.0 [5+]	58-77 8-57
CXCL8(9-77)	900.4 [9+]	615.3 [4+] 1128.7 [5+]	58-77 9-57

### Detection of CXCL8 Proteoforms in Cell Culture Supernatants From Human Osteosarcoma Cells Confirms Natural NH_2_-Terminal Heterogeneity

**Figure 3:**
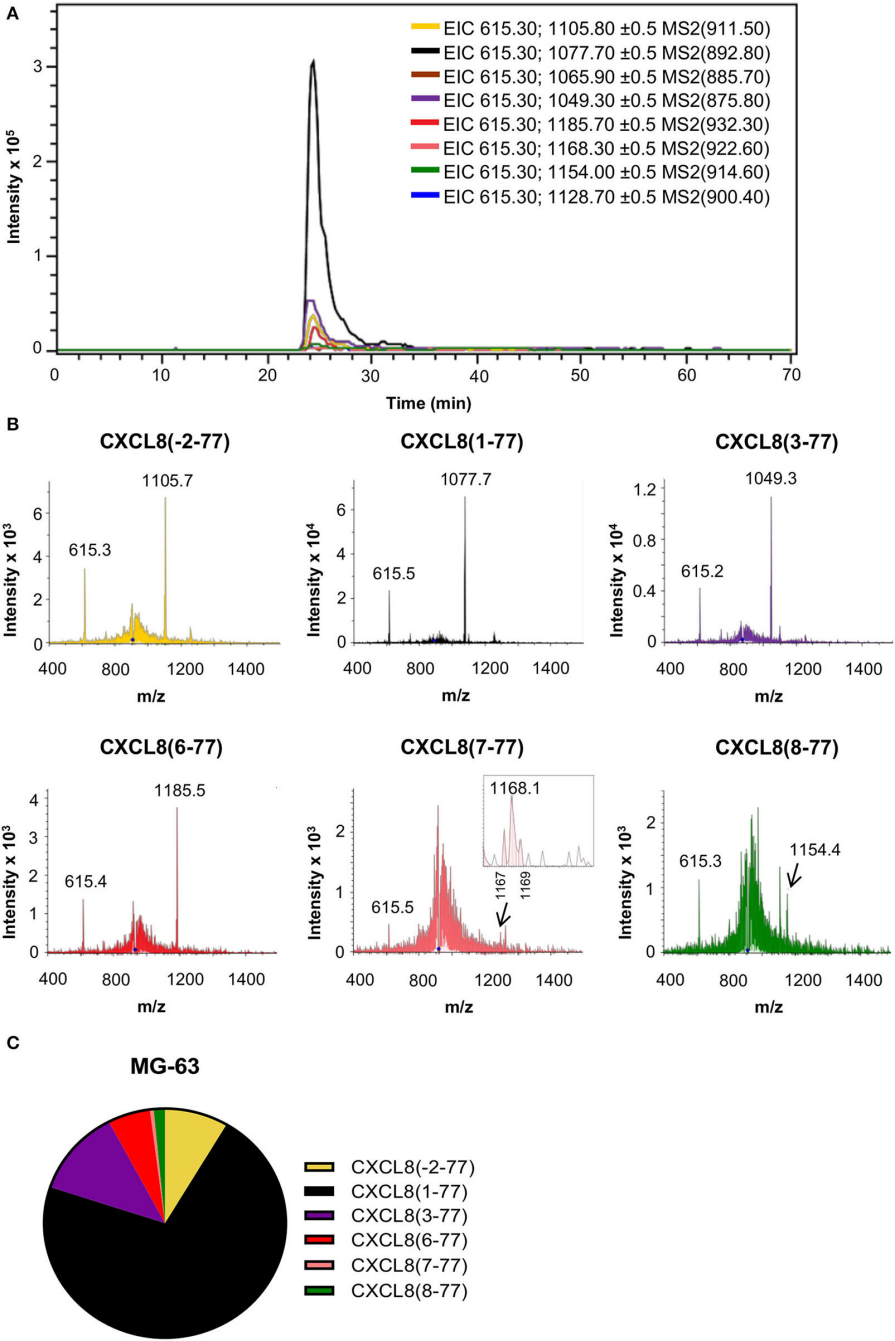
Detection of CXCL8 proteoforms in cell culture supernatant from osteosarcoma cells. The MG-63 osteosarcoma cell line was stimulated with the synthetic double stranded RNA poly rI:rC to produce CXCL8. Partially purified cell culture supernatant was subjected to top-down nano-LC-MS/MS analysis. Two nano-LC-MS/MS runs were performed to examine the presence and quantity of eight CXCL8 proteoforms in MRM mode. **(A)** Extracted ion chromatograms (EIC) showing the intensity of m/z values of signature fragment ions (±0.5) generated by fragmentation of a specific precursor ion (indicated between brackets) during protein elution. **(B)** Fragmentation spectra confirming the presence of signature ions of CXCL8(-2-77), CXCL8(1-77), CXCL8(3-77), CXCL8(6-77), CXCL8(7-77) and CXCL8(8-77). **(C)** Relative abundance of the detected CXCL8 proteoforms.

Large-scale purification of natural CXCL8 from diverse cellular sources including osteosarcoma cells (MG-63), fibroblasts, monocytes, endothelial and epithelial cells, revealed NH_2_-terminal sequence heterogeneity (15, 19, 30–34). To validate our nano-LC-MS/MS-based approach, we examined the presence of natural CXCL8 proteoforms in cell culture supernatant from osteosarcoma cells (MG-63) after partial purification by adsorption to CPG, heparin affinity and RP chromatography. As reported previously, osteosarcoma cell-derived CXCL8 was found to display NH_2_-terminal sequence heterogeneity (33, 34). Edman degradation was performed on 740 ng RP-HPLC purified CXCL8. Proteins corresponding to CXCL8(1-77) and CXCL8(3-77) with NH_2_-terminal sequences AVLPRSAKELRXQXIKTY and LPRSXKE were identified (X refers to an unidentified amino acid). A very weak signal of SX(K)(E)XRXQ, indicating the presence of CXCL8(6-77), was detected in which signals for Lys (K) and Glu (E) were close to the background. Analysis of EIC and fragmentation spectra, obtained after exposing a hundred-fold smaller amount of CXCL8 (7.4 ng) to nano-LC-MS/MS analysis, confirmed the occurrence of elongated CXCL8(-2-77), authentic CXCL8(1-77) and the truncated variants CXCL8(3-77), CXCL8(6-77), CXCL8(7-77), and CXCL8(8-77) (Figures 3A,B). Based on the intensity of their signature fragment ions, we found that CXCL8(1-77), representing 71.6% of the total amount of CXCL8, was the most abundant CXCL8 proteoform in supernatant of cultured MG-63 cells, followed by CXCL8(3-77) (12.2%), CXCL8(-2-77) (8.5%), and the highly potent truncated forms CXCL8(6-77) (5.7%), CXCL8(8-77) (1.5%), and CXCL8(7-77) (0.5%) (Figure 3C).

### Detection of Endogenous CXCL8 in Synovial Fluids From Arthritis Patients Proves Proteolytic Activation

Mounting evidence suggests a prominent role for neutrophils and neutrophil-attracting chemokines in chronic joint inflammation including RA and JIA (35–39). We collected synovial fluids from fourteen adult RA patients [median age (range) of 42 (22–79) years and female/male ratio of 8/6] and from twelve pediatric patients with oligoarticular (*n* = 9) or polyarticular (*n* = 3) JIA [median age (range) of 11 (4–17) years and female/male ratio of 9/3] to explore the potential role of CXCL8 processing in this disease context using our newly developed ISTAMPA technology. The workflow for this approach is depicted in Figure 1. To increase the sensitivity of the method and because viscous synovial fluids cannot be loaded onto a nano-LC column as such, a pre-purification step was required. To this end, for each patient sample, total CXCL8 was isolated from 0.5 ml of synovial fluid based on immunosorbent magnetic purification using biotinylated anti-CXCL8 antibodies coupled to streptavidin-labeled magnetic beads. Captured CXCL8 was recovered by elution in 20 μL 0.1 M Gly (pH 2.8), kept at 5°C in an autosampler and subjected to nano-LC-MS/MS analysis. We validated that the biotinylated anti–human CXCL8 antibody recognized intact and truncated CXCL8 bound to ELISA plates with similar efficiency (Supplementary Figure 3A). In addition, K_D_ values for intact CXCL8(1-77) (45.3 nM) and the shortest form CXCL8(9-77) (39.5 nM) were comparable on SPR. Moreover, analysis of healthy human plasma containing equal amounts (5 ng) of exogeneous CXCL8(1-77), CXCL8(6-77) and CXCL8(9-77) by ISTAMPA confirmed the successful recovery and relative quantification of intact and truncated CXCL8 proteoforms (Supplementary Figures 3B,C).

**Figure 4:**
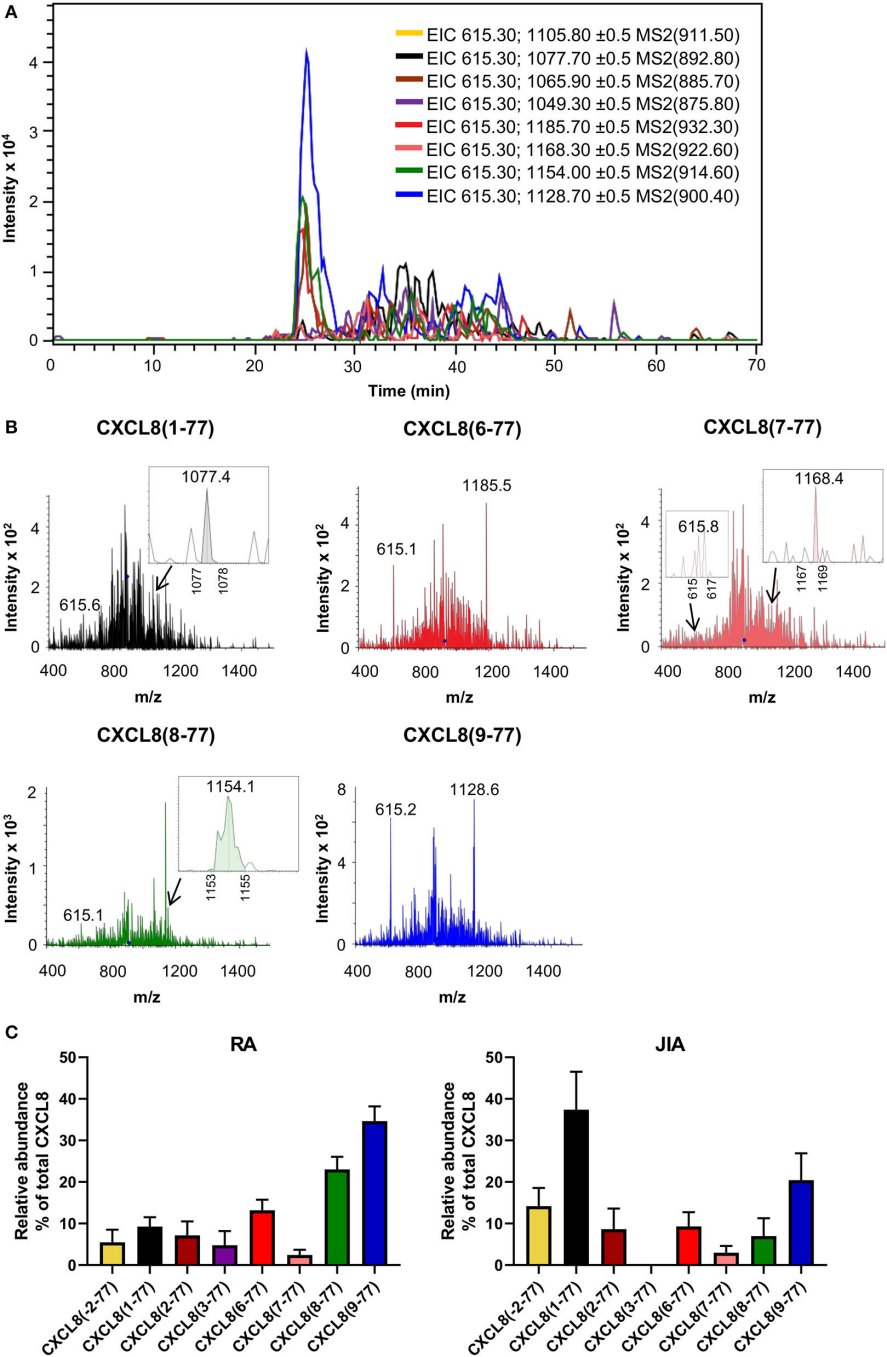
Detection of CXCL8 by top down-tandem mass spectrometry proves proteolytic activation in synovial fluids of arthritis patients. Total CXCL8 was extracted from synovial fluids of RA (*n* = 14) and JIA patients (*n* = 12) by immunosorbent isolation and subjected to nano-LC-MS/MS analysis. For each sample, two top-down nano-LC-MS/MS runs were performed to examine the presence of eight CXCL8 forms in MRM mode. **(A)** Extracted ion chromatograms (EIC) show the intensity of m/z values of signature fragment ions (±0.5) generated by fragmentation of a specific precursor ion (indicated between brackets) during protein elution. A representative experiment is shown (RA patient). **(B)** Representative fragmentation spectra confirm the presence of signature ions of CXCL8(1-77), CXCL8(6-77), CXCL8(7-77), CXCL8(8-77) and CXCL8(9-77) in synovial fluid from a patient with RA. **(C)** Relative abundance of CXCL8 proteoforms in synovial fluids from RA and JIA patients (represented as mean ± SEM).

Total CXCL8 levels in synovial fluids from RA and JIA patients were 19.5 ± 6.9 ng/ml (mean ± SEM) and 1.8 ± 0.7 ng/ml (mean ± SEM), respectively, as determined by ELISA. Analysis of fragmentation spectra uncovered that, on average, intact CXCL8(1-77) accounted for only 9.3 ± 2.3% (mean ± SEM) of total CXCL8 in synovial fluids from RA patients. Approximately 85% of synovial fluid-derived CXCL8 from RA patients was NH_2_-terminally truncated. Strikingly, the shortest CXCL8 proteins, i.e., CXCL8(8-77) and CXCL8(9-77) which are known to possess up to 30-fold enhanced activity as compared to full-length CXCL8(1-77), were the most abundant CXCL8 proteoforms in synovial fluids from RA patients, representing 23.0 ± 3.0% (mean ± SEM) and 34.6 ± 3.6% (mean ± SEM) of total synovial CXCL8, respectively (Figure 4; Supplementary Table 1). In addition, CXCL8(6-77) was detected in most RA synovial fluids [13.2 ± 2.5% (mean ± SEM)] (Figures 4A–C; Supplementary Table 1). A few RA synovial fluids contained detectable levels of CXCL8(-2-77), CXCL8(2-77), CXCL8(3-77) and/or CXCL8(7-77) (Supplementary Table 1). As compared to RA, synovial fluids from JIA patients contained higher percentages of intact CXCL8. The relative abundance of intact CXCL8(1-77) and elongated CXCL8(-2-77) in JIA synovial fluids was 37.4 ± 9.1% (mean ± SEM) and 14.2 ± 4.4% (mean ± SEM), respectively. The most prominent truncated proteoform in synovial fluid from JIA patients was the most active CXCL8 protein CXCL8(9-77) [20.4 ± 6.5% (mean ± SEM)], followed by CXCL8(6-77) [9.3 ± 3.4% (mean ± SEM)], CXCL8(2-77) [8.6 ± 5.0% (mean ± SEM)], CXCL8(8-77) [7.0 ± 4.3% (mean ± SEM)] and CXCL8(7-77) [3.0 ± 1.6% (mean ± SEM)] (Figure 4C; Supplementary Table 1). Results from five independent measurements of a single RA sample confirmed the reproducibility of the method if low CXCL8 concentrations are present (Supplementary Figure 4).

### Kinetics of CXCL8 Processing in the Presence of Synovial Fluids

**Figure 5:**
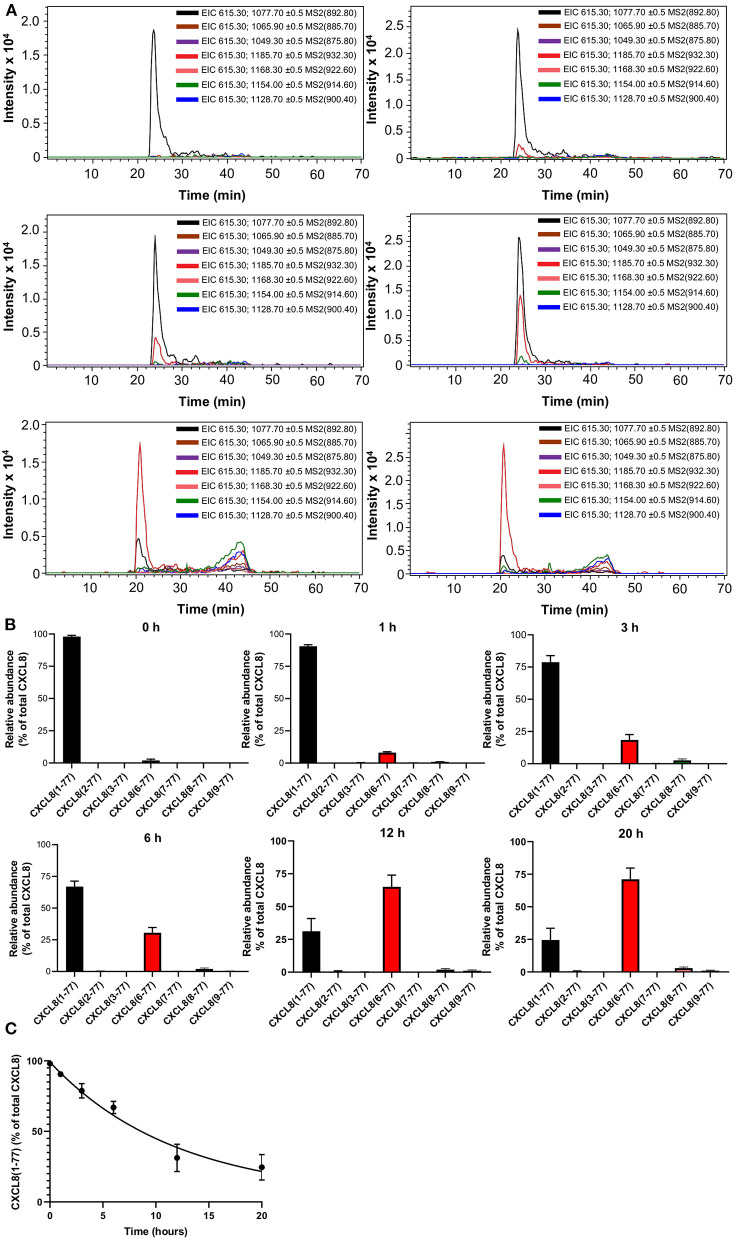
Kinetics of CXCL8 processing in the presence of synovial fluids from juvenile idiopathic arthritis patients. Exogenous CXCL8(1-77) was incubated with synovial fluids from JIA patients (*n* = 4) for a period of 0, 1, 3, 6, 12 or 20 h. Total CXCL8 was extracted from synovial fluids by immunosorbent isolation and subjected to nano-LC-MS/MS analysis. For each sample, two nano-LC-MS/MS runs were performed to examine the presence of eight CXCL8 forms in MRM mode. **(A)** Extracted ion chromatograms (EIC) show the intensity of m/z values of signature fragment ions (±0.5) generated by fragmentation of a specific precursor ion (indicated between brackets) during protein elution. **(B)** Relative abundance of CXCL8 proteoforms in synovial fluids after 0, 1, 3, 6, 12, and 20 h of incubation (represented as mean ± SEM). **(C)** Pseudo-first order association kinetics of proteolytic modification of CXCL8(1-77) in the presence of synovial fluids (represented as mean ± SEM; *n* = 4).

To investigate the kinetics of CXCL8 truncation in the presence of synovial fluids of arthritis patients, we exposed exogeneous CXCL8(1-77) to a small volume (20 μL, which is too small to detect endogeneous CXCL8 proteoforms) of synovial fluids from JIA patients (*n* = 4). As expected, CXCL8(1-77) was the most abundant CXCL8 proteoform if samples were immediately purified by immunosorbent isolation and analyzed with nano-LC-MS/MS (Figures 5A,B; Supplementary Figure 5A; Supplementary Table 2). After 1-20 h of incubation, full-length CXCL8(1-77) was progressively processed to NH_2_-terminally truncated, more potent proteoforms. The most frequently measured CXCL8 proteoform in incubation mixtures was CXCL8(6-77), accounting for 8.1 ± 0.6% (mean ± SEM), 18.5 ± 4.1% (mean ± SEM), 30.5 ± 4.1% (mean ± SEM), 65.1 ± 8.8% (mean ± SEM) and 71.1 ± 8.7% (mean ± SEM) of total CXCL8 after 1, 3, 6, 12, and 20 h, respectively (Figures 5A,B; Supplementary Figures 5B–D; Supplementary Table 2). In addition, minute amounts of CXCL8(8-77) and CXCL8(9-77) were detected if CXCL8(1-77) was exposed to JIA synovial fluids. The relative abundance of CXCL8(8-77) after 1, 3, 6, 12, and 20 h was 1.0 ± 0.4% (mean ± SEM), 2.6 ± 1.0% (mean ± SEM), 2.0 ± 0.8% (mean ± SEM), 2.0 ± 0.8% (mean ± SEM) and 3.0 ± 0.9% (mean ± SEM), respectively (Figures 5A,B; Supplementary Table 2). CXCL8(9-77) represented 1.2 ± 0.6% (mean ± SEM) and 1.0 ± 0.4% (mean ± SEM) of total CXCL8 after 12 and 20 h, respectively (Supplementary Figures 5E,F). The half-life of intact CXCL8(1-77) in the presence of synovial fluids from JIA patients was 8.2 h (Figure 5C).

## Discussion

The significance of posttranslational modifications has been evidenced in various immunological settings. For example, caspase-mediated proteolytic activation is a prerequisite for generation of biologically active IL-1β and IL-18 (40, 41). Protein citrullination, presumably, plays an important role in the pathogenesis of multiple sclerosis and RA (42–44). Intense former research efforts demonstrated the potentially substantial effects of posttranslational modifications on the biological activity and receptor preferences of chemokines *in vitro* and *in vivo* [reviewed in (6, 10)]. However, their importance in patient samples is unclear due to a lack of specific detection methods for proteoforms with different activities. In the past, we purified multiple naturally processed chemokines from cell culture supernatants using a multi-step analytical purification method that combines (1) adsorption to CPG, (2) antibody or heparin-Sepharose affinity chromatography, (3) cation exchange chromatography and (4) RP-HPLC and confirmed protein identity by Edman degradation (20, 33). Though proven successful, this time-consuming analytical approach requires liters of starting material. The lack of more straightforward immuno-assays able to distinguish differentially processed chemokines and the limited volumes of clinical samples available for research purposes hamper detection of distinct chemokine forms in human body fluids.

Despite the difficulties that challenge detection and quantification of chemokine proteoforms with different activities in biological samples, a few scientific reports validating the occurrence of differentially processed chemokines *in vivo* were published. Consequently, the role of posttranslational modifications in fine-tuning the precise chemokine activity can no longer be neglected. Proteoforms of CXCL10 and CXCL12 that lack two NH_2_-terminal residues as a result of CD26-mediated proteolysis, have been detected in human plasma (45, 46). Moreover, an emerging body of evidence supports the notion that interfering with chemokine processing may have therapeutic benefits. For example, *in vivo* inhibition of the protease CD26 resulted in elevated concentrations of intact CXCL10 accompanied by enhanced anti-tumor activity in mice (7). Likewise, inhibition of CD26 results in enhanced availability of biologically active CXCL12, thereby improving wound healing (8). Inhibition of the isoenzyme of glutaminyl cyclase that is responsible for cyclization of glutamine into pyroglutamic acid, coincided with reduction of CCL2-driven monocyte recruitment *in vivo* with beneficial effects on atherosclerosis progression (47).

In the present study, we introduce in an exemplary way a straightforward, approach for simultaneous detection and relative quantification of differentially processed proteoforms of the aglycosyl chemokine CXCL8. Following immunosorbent sample preparation, top-down ion trap tandem mass spectrometry with limited fragmentation energy is exploited for identification of native CXCL8(1-77), elongated CXCL8(-2-77), and naturally occurring truncated CXCL8 proteoforms lacking one to eight NH_2_-terminal amino acids. Considering that we aimed to detect specific proteoforms with minimal structural differences, a major advantage of our top-down approach over commercially available bottom-up specific enrichment methods such as “stable isotope standards and capture by anti-peptide antibodies” (SISCAPA) is the fact that there is no requirement for initial enzymatic protein digestion and/or stable-isotope labeling (48). Indeed, protein digestion by proteases such as trypsin, the preferred enzyme in bottom-up proteomics, will cleave NH_2_-terminal peptides from chemokines and prevent correct relative quantification of NH_2_-terminal proteoforms known to have different activities. It is generally acknowledged that information on the occurrence of specific proteoforms of the molecule of interest gets lost easily upon protein digestion used in bottom-up proteomics (49, 50). Furthermore, anti-peptide specific antibodies cannot be used to discriminate between chemokine proteoforms. Although marking peptides with stable isotopes may improve the sensitivity of mass spectrometry-based methods for protein quantification and reduces experimental bias, working with stable isotope-labeling is less practical, implies a higher cost and the need of larger amounts of starting material as compared to label-free approaches (51). Moreover, sample preparation and data analysis are more complex.

In contrast to mass spectrometric immunoassays (MSIA), the ISTAMPA procedure can be performed with a standard electrospray ionization – ion trap mass spectrometer. Therefore, this method is accessible to any laboratory well equipped for standard tandem mass spectrometry. The generation of only two signature fragment ions using limited fragmentation energy ensures maximal sensitivity of the method. Our method was validated in patient samples and we confirmed the existence of natural NH_2_-terminally cleaved CXCL8 proteoforms in synovial fluids from arthritis patients. Although oligoarticular/polyarticular JIA and RA were traditionally considered to be antigen-driven autoimmune diseases with a predominant role for T cells, the complete spectrum of immune-inflammatory responses as seen in patients probably depends on complex interactions between innate and adaptive immunity. An important role for neutrophils, acting at the crossroads of innate and adaptive immunity, in synovial inflammation characteristic of JIA and RA has been speculated (35, 36, 38, 39, 52). Detection of CXCL8 proteoforms with superior potencies in synovial fluids from patients favors this idea and opens a window of opportunity for identification of potential drug targets. Importantly, neutrophils may promote their own recruitment and activation by secreting enzymes known to process CXCL8 into more potent, truncated proteoforms to set up an auto-amplification loop of neutrophilic inflammation, eventually leading to tissue damage if patients are not treated appropriately (24).

## Data Availability Statement

The original data presented in this study are publicly available. Data can be found here: [ftp://massive.ucsd.edu/MSV000086840/]. Other data are included in the article or Supplementary Materials.

## Ethics Statement

The studies involving human participants were reviewed and approved by Ethics Committee of the University Hospital Leuven. Written informed consent to participate in this study was provided by the participants' legal guardian/next of kin.

## Author Contributions

BN, PV, LD, and CW were responsible for diagnosis and recruitment of patients. MM, SA, JVa, AM, RJ, SV, EG, EM, GO, and PP performed experiments and analyzed data. PP supervised the study. MM wrote the initial manuscript with assistance of SA. All authors contributed to the study conception and design, commented on previous versions of the manuscript and approved the final version of the manuscript.

## Conflict of Interest

The authors declare that the research was conducted in the absence of any commercial or financial relationships that could be construed as a potential conflict of interest.
