# Supplementary material for: From ELISA to Immunosorbent Tandem Mass Spectrometry Proteoform Analysis: The Example of CXCL8/Interleukin-8
Source: Front Immunol. 2021 Mar 11;12:644725. doi: 10.3389/fimmu.2021.644725 (PMC7991300; doi:10.3389/fimmu.2021.644725)
Supplement: Supplementary file 1 [file Data_Sheet_1.PDF]

From ELISA to immunosorbent tandem mass spectrometry proteoform analysis (ISTAMPA): the example of CXCL8/interleukin-8

Mieke Metzemaekers[1], Sara Abouelasrar Salama[1], Jennifer Vandooren[2], Anneleen Mortier[1], Rik Janssens[1], Sofie Vandendriessche[1], Eva Ganseman[1], Erik Martens[2], Mieke Gouwy[1], Barbara Neerinckx[3], Patrick Verschueren[3], Lien De Somer[2], Carine Wouters[2], Sofie Struyf[1], Ghislain Opdenakker[2], Jo Van Damme[1] and Paul Proost[1,*]

[1]Laboratory of Molecular Immunology, Rega Institute, Department of Microbiology, Immunology and Transplantation, KU Leuven, Leuven, Belgium

[2]Laboratory of Immunobiology, Rega Institute, Department of Microbiology, Immunology and Transplantation, KU Leuven, Leuven, Belgium

[3]Skeletal Biology and Engineering Research Center, Department of Development and Regeneration, KU Leuven, Leuven, Belgium

*** Correspondence:**
Paul Proost
paul.proost@kuleuven.be

Supplementary Material

Supplementary Table S1. Relative abundance of endogenous CXCL8 forms in synovial fluids from RA and JIA patients.

Patiënt	Synovial CXCL8 concentration (ng/ml)	CXCL8(-2-77) (%)	CXCL8(1-77) (%)	CXCL8(2-77) (%)	CXCL8(3-77) (%)	CXCL8(6-77) (%)	CXCL8(7-77) (%)	CXCL8(8-77) (%)	CXCL8(9-77) (%)
**RA1**	13,9	N.D.	17,5	N.D.	N.D.	24,6	N.D.	18,4	39,5
**RA2**	2,9	N.D.	N.D.	38,8	N.D.	12,9	N.D.	17,9	30,4
**RA3**	8,2	N.D.	14,4	18,5	N.D.	8,7	10,0	25,8	22,6
**RA4**	27,6	23,1	9,3	N.D.	N.D.	9,3	6,7	26,6	25,0
**RA5**	5,8	N.D.	N.D.	N.D.	N.D.	15,1	14,3	17,0	53,5
**RA6**	99,4	3,2	3,3	N.D.	N.D.	18,6	2,6	24,2	48,1
**RA7**	18,7	N.D.	9,7	24,1	N.D.	27,0	N.D.	15,3	23,9
**RA8**	48,0	N.D.	11,8	N.D.	N.D.	13,7	0,8	26,8	47,0
**RA9**	7,3	N.D.	23,1	N.D.	23,2	N.D.	N.D.	28,7	25,0
**RA10**	12,6	N.D.	6,7	N.D.	N.D.	17,6	N.D.	15,3	60,5
**RA11**	4,8	13,9	8,8	N.D.	N.D.	10,0	N.D.	52,0	15,2
**RA12**	8,4	36,6	N.D.	N.D.	N.D.	27,4	N.D.	N.D.	35,9
**RA13**	13,6	N.D.	N.D.	N.D.	43,5	N.D.	N.D.	24,6	31,9
**RA14**	2,4	N.D.	25,0	18,8	N.D.	N.D.	N.D.	29,8	26,4
**JIA1**	0,4	33,5	48,0	N.D.	N.D.	18,5	N.D.	N.D.	N.D.
**JIA2**	0,6	11,8	27,0	N.D.	N.D.	N.D.	8,7	N.D.	52,4
**JIA3**	0,4	N.D.	29,4	N.D.	N.D.	34,5	N.D.	N.D.	36,1
**JIA4**	0,2	N.D.	33,3	54,7	N.D.	N.D.	12,0	N.D.	N.D.
**JIA5**	1,0	16,2	22,6	25,9	N.D.	24,4	N.D.	N.D.	10,8
**JIA6**	1,4	50,0	N.D.	N.D.	N.D.	N.D.	N.D.	N.D.	50,0
**JIA7**	1,8	10,9	25,7	N.D.	N.D.	16,1	15,2	18,2	13,8
**JIA8**	8,3	17,6	24,5	N.D.	N.D.	12,0	N.D.	17,0	29,0
**JIA9**	5,0	N.D.	100,0	N.D.	N.D.	N.D.	N.D.	N.D.	N.D.
**JIA10**	1,1	12,7	9,3	22,9	N.D.	6,3	N.D.	48,7	N.D.
**JIA11**	0,7	17,7	29,1	N.D.	N.D.	N.D.	N.D.	N.D.	53,1
**JIA12**	0,3	N.D.	100,0	N.D.	N.D.	N.D.	N.D.	N.D.	N.D.

Abbreviations: JIA, juvenile idiopathic arthritis; N.D., not detected; RA, rheumatoid arthritis.

Supplementary Table S2. Kinetics of CXCL8 processing in the presence of synovial fluid.

Patient	Duration of incubation (hours)	CXCL8(1-77) (%)	CXCL8(2-77) (%)	CXCL8(3-77) (%)	CXCL8(6-77) (%)	CXCL8(7-77) (%)	CXCL8(8-77) (%)	CXCL8(9-77) (%)
**JIA1**	0	98,3	N.D.	N.D.	1,7	N.D.	N.D.	N.D.
**JIA2**	0	98,4	N.D.	N.D.	1,6	N.D.	N.D.	N.D.
**JIA3**	0	100,0	N.D.	N.D.	N.D.	N.D.	N.D.	N.D.
**JIA4**	0	95,4	N.D.	N.D.	4,6	N.D.	N.D.	N.D.
**JIA1**	1	91,8	N.D.	N.D.	7,1	N.D.	1,1	N.D.
**JIA2**	1	89,3	N.D.	N.D.	9,0	N.D.	1,7	N.D.
**JIA3**	1	93,0	N.D.	N.D.	7,0	N.D.	N.D.	N.D.
**JIA4**	1	88,2	N.D.	0,7	9,4	0,7	1,0	N.D.
**JIA1**	3	66,2	N.D.	N.D.	28,7	N.D.	5,1	N.D.
**JIA2**	3	80,1	N.D.	N.D.	17,3	N.D.	2,6	N.D.
**JIA3**	3	91,0	N.D.	N.D.	8,5	0,5	N.D.	N.D.
**JIA4**	3	77,9	N.D.	N.D.	19,4	N.D.	2,8	N.D.
**JIA1**	6	59,7	N.D.	N.D.	39,2	N.D.	1,1	N.D.
**JIA2**	6	61,9	N.D.	N.D.	33,8	N.D.	4,3	N.D.
**JIA3**	6	79,1	N.D.	N.D.	19,5	N.D.	0,8	0,6
**JIA4**	6	67,2	0,5	N.D.	29,5	0,5	1,8	0,5
**JIA1**	12	23,6	N.D.	N.D.	74,7	N.D.	0,6	1,1
**JIA2**	12	19,3	1,9	N.D.	72,3	N.D.	3,8	2,8
**JIA3**	12	60,1	N.D.	N.D.	38,6	N.D.	0,8	0,5
**JIA4**	12	21,7	0,4	0,4	74,7	N.D.	2,6	0,2
**JIA1**	20	16,5	N.D.	N.D.	82,2	N.D.	0,8	0,5
**JIA2**	20	11,3	1,2	N.D.	80,2	N.D.	5,0	2,2
**JIA3**	20	51,1	N.D.	N.D.	45,4	N.D.	2,9	0,6
**JIA4**	20	19,1	0,8	N.D.	76,6	N.D.	3,1	0,4

Abbreviations: JIA, juvenile idiopathic arthritis; N.D., not detected.

$_{-2}$EGAVLPRSAKELRCQCIKTYSKPFHPKFIKELRVIESGPHCANTEIIVKLSDGRELCL **DP** KENWVQRVVEKFLKRAENS$_{77}$ **CXCL8(-2-77)**

$_{1}$AVLPRSAKELRCQCIKTYSKPFHPKFIKELRVIESGPHCANTEIIVKLSDGRELCL **DP** KENWVQRVVEKFLKRAENS$_{77}$ **CXCL8(1-77)**

$_{2}$VLPRSAKELRCQCIKTYSKPFHPKFIKELRVIESGPHCANTEIIVKLSDGRELCL **DP** KENWVQRVVEKFLKRAENS$_{77}$ **CXCL8(2-77)**

$_{3}$LPRSAKELRCQCIKTYSKPFHPKFIKELRVIESGPHCANTEIIVKLSDGRELCL **DP** KENWVQRVVEKFLKRAENS$_{77}$ **CXCL8(3-77)**

$_{6}$SAKELRCQCIKTYSKPFHPKFIKELRVIESGPHCANTEIIVKLSDGRELCL **DP** KENWVQRVVEKFLKRAENS$_{77}$ **CXCL8(6-77)**

$_{7}$AKELRCQCIKTYSKPFHPKFIKELRVIESGPHCANTEIIVKLSDGRELCL **DP** KENWVQRVVEKFLKRAENS$_{77}$ **CXCL8(7-77)**

$_{8}$KELRCQCIKTYSKPFHPKFIKELRVIESGPHCANTEIIVKLSDGRELCL **DP** KENWVQRVVEKFLKRAENS$_{77}$ **CXCL8(8-77)**

$_{9}$ELRCQCIKTYSKPFHPKFIKELRVIESGPHCANTEIIVKLSDGRELCL **DP** KENWVQRVVEKFLKRAENS$_{77}$ **CXCL8(9-77)**

Supplementary Figure S1. Protein sequence of CXCL8 forms. Natural CXCL8 displays NH_2-terminal heterogeneity. Most CXCL8 forms are generated by site-specific proteolytic modification of full-length CXCL8(1-77). In addition, an elongated form was described, *i.e.* CXCL8(-2-77), that presumably results from alternative splicing of the signal peptide.

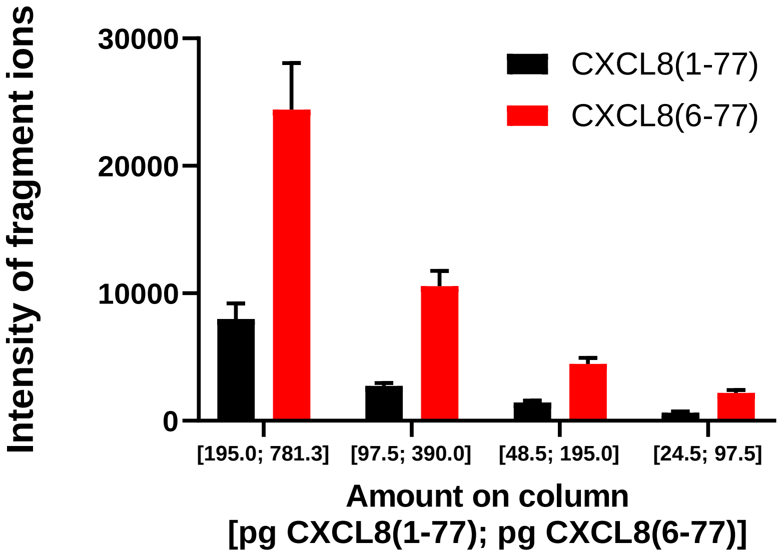

Supplementary Figure S2. Simultaneous quantification of CXCL8 forms by ion trap tandem mass spectrometry. Analysis of a mixture containing CXCL8(1-77) and CXCL8(6-77) (ratio 1:4) by nano-LC-MS/MS. CXCL8 forms were quantified based on the intensity of their signature fragment ions. Results are represented as mean ± SEM (n = 5).

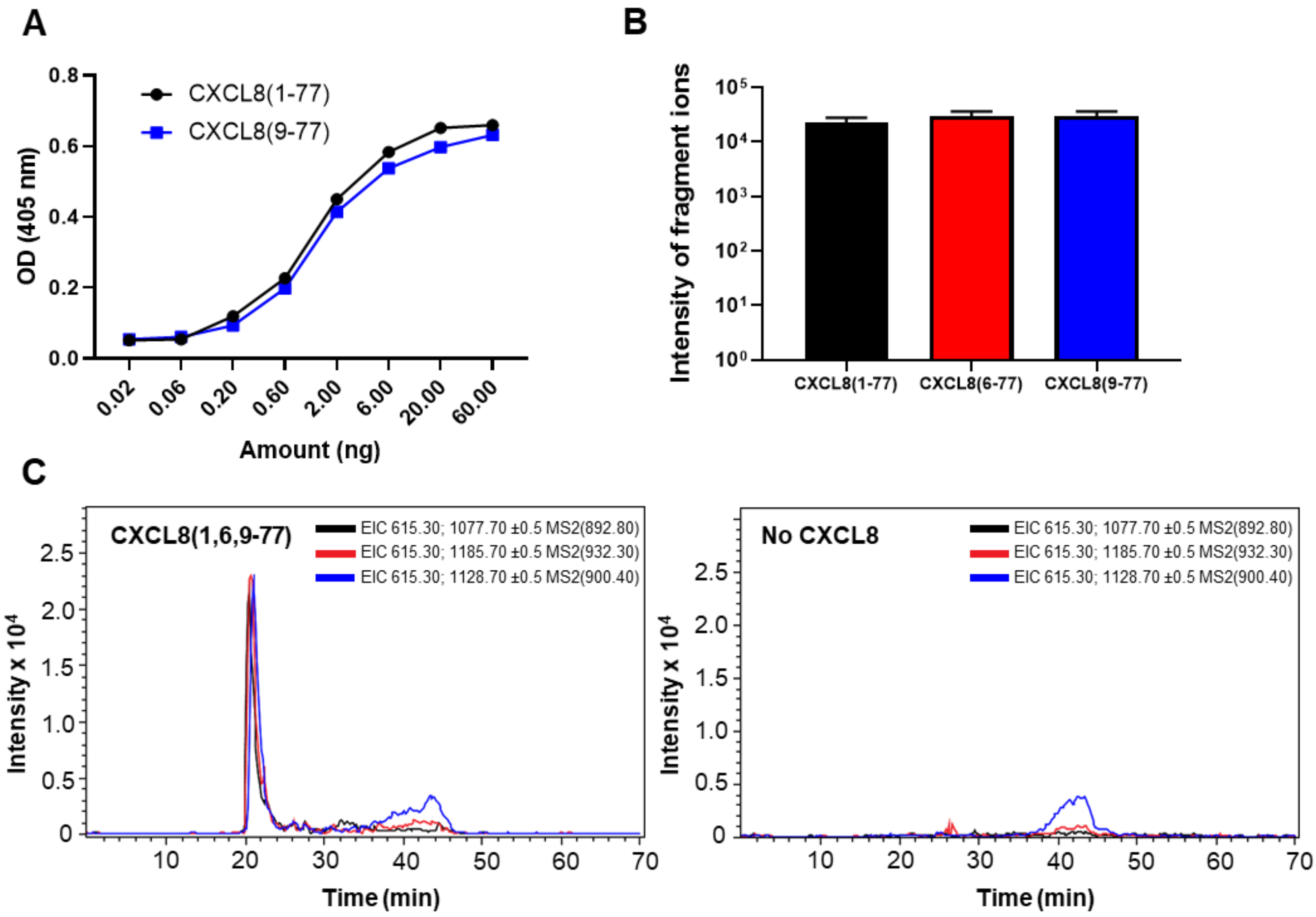

Supplementary Figure S3. Capturing of CXCL8 forms by polyclonal rabbit anti-human CXCL8. (A) CXCL8 forms were immobilized on a 96-well plate and detected with biotinylated anti-human CXCL8 (6.7 ng/well) and peroxidase-conjugated streptavidin. Absorbance values were measured at 450 nm. (B-C) Plasma from a healthy human volunteer was enriched with CXCL8(1-77), CXCL8(6-77) and CXCL8(9-77) (5 ng of each form). Total CXCL8 was extracted by immunosorbent isolation and subjected to nano-LC-MS/MS analysis. (B) CXCL8 forms were quantified based on the intensity of their signature fragment ions at the elution time of CXCL8. Results are represented as mean ± SEM (n = 4). (C) Extracted ion chromatograms (EIC) show the intensity of m/z values of signature fragment ions (±0.5) generated by fragmentation of a specific precursor ion (indicated between brackets) during protein elution. A representative experiment is shown (n = 4). A negative control (plasma without CXCL8) is depicted on the right part of the figure.

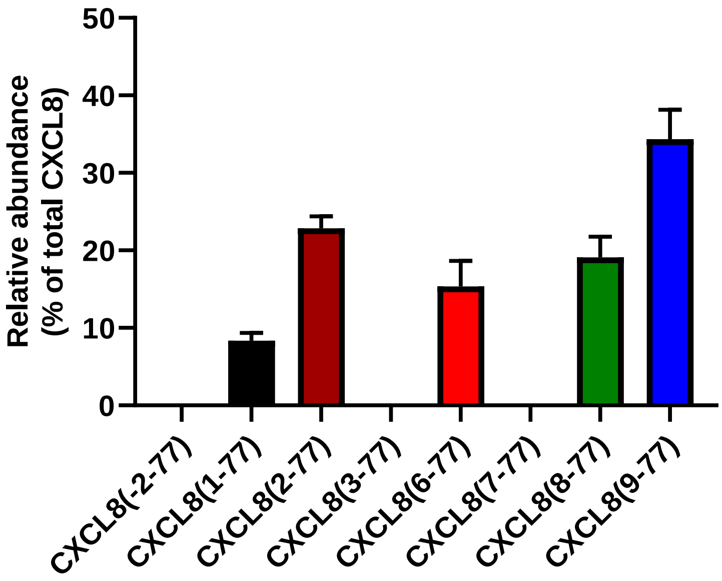

Supplementary Figure S4. Reproducibility of CXCL8 proteoform quantification in synovial fluid by ISTAMPA. Total CXCL8 was extracted from synovial fluid of a RA patient by immunosorbent isolation and subjected to nano-LC-MS/MS analysis. Five independent relative quantifications of CXCL8 proteoforms of the same sample were performed. The presence of eight CXCL8 forms was assessed by nano-LC-MS/MS in MRM mode. Figure shows the relative abundance of CXCL8 proteoforms (represented as mean ± SEM) (n = 5).

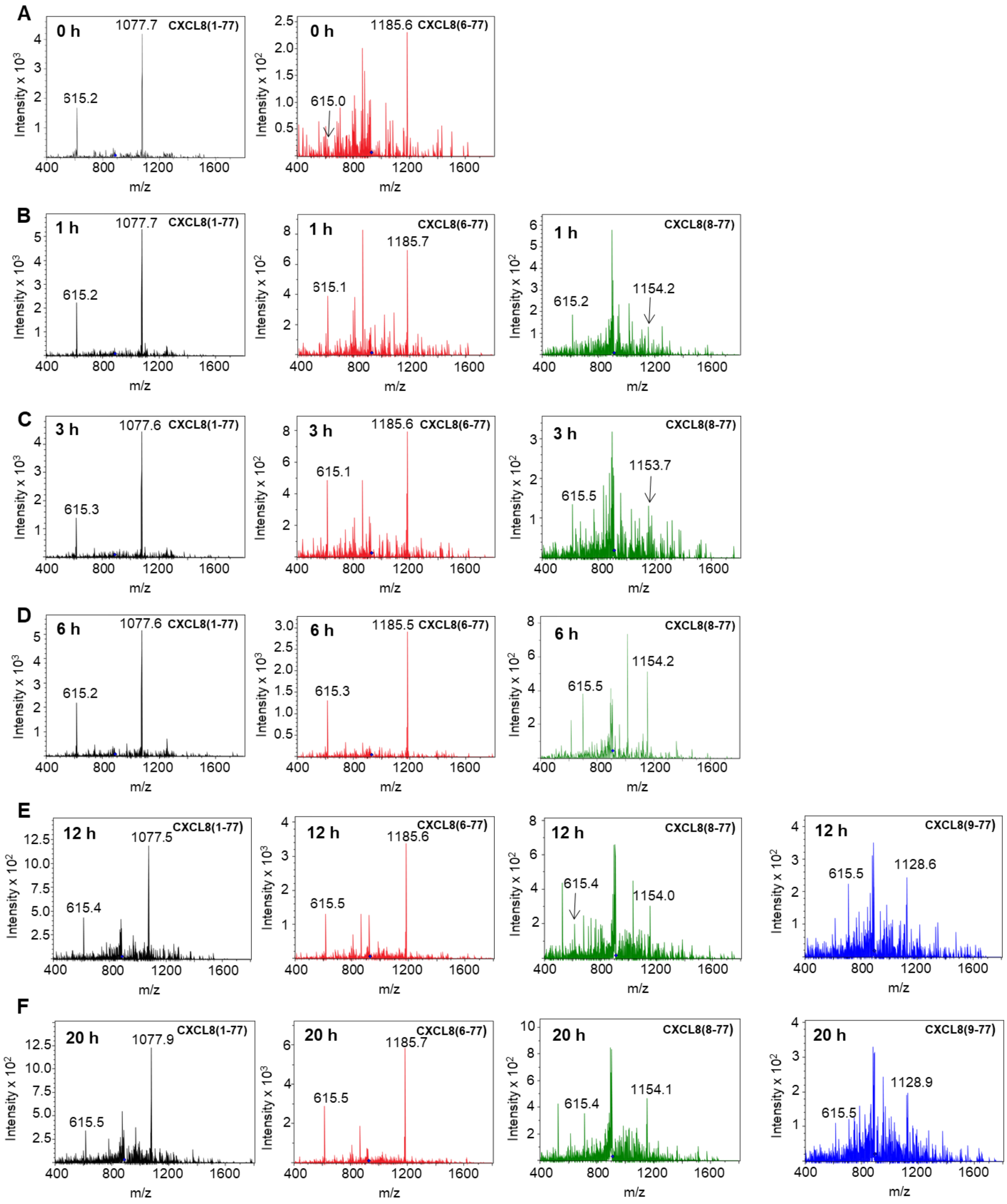

Supplementary Figure S5. Kinetics of CXCL8 processing in the presence of synovial fluids from juvenile idiopathic arthritis patients. Exogenous CXCL8(1-77) was incubated with synovial fluids from JIA patients (n = 4) for a period of 0, 1, 3, 6, 12 or 20 hours. Total CXCL8 was extracted from

synovial fluids by immunomagnetic isolation and subjected to nano-LC-MS/MS analysis. For each sample, two nano-LC-MS/MS runs were performed to examine the presence of eight CXCL8 forms in MRM mode. Representative fragmentation spectra confirming the presence of signature ions of CXCL8(1-77), CXCL8(6-77) and CXCL8(8-77) after 0, 1, 3, 6, 12 and 20 hours of incubation are shown in panel A, B, C, D, E and F, respectively.
